# Functional and genetic analysis of the colon cancer network

**DOI:** 10.1186/1471-2105-15-S6-S6

**Published:** 2014-05-16

**Authors:** Frank Emmert-Streib, Ricardo de Matos Simoes, Galina Glazko, Simon McDade, Benjamin Haibe-Kains, Andreas Holzinger, Matthias Dehmer, Frederick Charles Campbell

**Affiliations:** 1 Computational Biology and Machine Learning Laboratory, Center for Cancer Research and Cell Biology, School of Medicine, Dentistry and Biomedical Sciences, Faculty of Medicine, Health and Life Sciences, Queen's University Belfast, 97 Lisburn Road, Belfast BT9 7BL, UK; 2 Division of Biomedical Informatics, University of Arkansas for Medical Sciences, Little Rock, AR 72205, USA; 3 Center for Cancer Research and Cell Biology, School of Medicine, Dentistry and Biomedical Sciences, Faculty of Medicine, Health and Life Sciences, Queen's University Belfast, 97 Lisburn Road, Belfast BT9 7BL, UK; 4 Bioinformatics and Computational Genomics Laboratory, Princess Margaret Cancer Centre, University of Toronto, Department of Medical Biophysics, Canada; 5 Institute for Medical Informatics, Statistics and Documentation, Medical University Graz, Auenbruggerplatz 2, 8036 Graz, Austria; 6 Institute for Bioinformatics and Translational Research, UMIT, Eduard Wallnoefer Zentrum 1, 6060, Hall in Tyrol, Austria

## Abstract

Cancer is a complex disease that has proven to be difficult to understand on the single-gene level. For this reason a functional elucidation needs to take interactions among genes on a systems-level into account. In this study, we infer a colon cancer network from a large-scale gene expression data set by using the method BC3Net. We provide a structural and a functional analysis of this network and also connect its molecular interaction structure with the chromosomal locations of the genes enabling the definition of cis- and trans-interactions. Furthermore, we investigate the interaction of genes that can be found in close neighborhoods on the chromosomes to gain insight into regulatory mechanisms. To our knowledge this is the first study analyzing the genome-scale colon cancer network.

## Background

Colon cancer is one of the leading causes of cancer related mortality in the western world [1]. It is a complex disease that is thought to mainly arise from polypoid lesions in the intestines as a result of inherited or somatic genetic alterations. These precursor lesions acquire further aberrations as they progress from adenoma to adenocarcinoma to metastatic disease, which in a simplified view can be described as a successive cascade of genetic changes [2,3]. The most common gene mutations occurring in colorectal cancer effect *APC *(tumor supressor), *MLH1, TP53, SMAD4, KRAS *and *BRAF *[4]. While significant progress has recently been made in characterizing the heterogeneity of the resulting disease subtypes and the effects of different combinations of these common mutations, a better understanding of the underlying gene networks is required, particularly, since the identification of general biomarkers has been unsuccessful as the disease stages and forms are highly specific to individuals. One reason for this observation is that genes are organized in non-linear overlapping pathways and act in a complex cellular network. Such an organizational structure allows alternative regulatory mechanisms to differentially control similar biological processes. Hence, multiple combinations of genes can result in similar phenotypic outcomes. As a result, cancer can be considered a pathway disease, which cannot be well characterized by individual marker genes [5,6]. For example, in colorectal cancer, activation of Wnt signaling is observed in nearly all tumors. However this can be mediated by inactivating mutation of the APC gene or hyper-activation of beta-catenin, or through mutation of genes with functions analogous to APC [7].

Due to experimental limitations, our knowledge of the underlying network in the cancer specific context is limited. Rather gene regulatory networks are inferred from large-scale gene expression data and provide a description of the mutual dependency structure between individual genes. The relationships represent different interaction types within the gene network that involve transcriptional regulatory interactions, (e.g. transcription factor target gene interactions); protein-protein interactions (e.g. between units of a protein complex) or more transient protein modifying interactions (e.g. phosphorylation events).

There are many factors that are thought to influence the regulation and explain changes of gene expression or signaling pathways that govern growth and differentiation processes. In sporadic colon cancer chromosomal instability [8] and microsatellite instability have been well described as phenotypes associated with subclasses of tumor types. In addition, epigenetic alterations such as methylation that affect gene expression of genes responsible for processes related to cancer progression have been shown to play important roles in disease development and progression [9]. Consequently, genetic and epigenetic events can lead to deregulation of multiple adjacent genes. For example, overexpression of multiple genes on Chromosome 13q is frequently observed in colorectal cancer [10-14].

In our study, we perform a systems analysis of the colon cancer gene regulatory network with respect to functional properties of the network structure and known cancer genes. To this end, we infer a BC3Net [15] gene regulatory network from a large-scale colon cancer gene expression data set (*GSE2109*) provided by the International Genomics Consortium (IGC). Furthermore, we explore the role of interactions between genes co-located on the same or on different chromosomes. We call these different interaction types cis- and trans-interactions. Finally, we study close neighborhoods on the chromosomes with respect to the connectivity of genes they contain as well as their biological function. The goal of our study is to identify and analyze co-regulated subnetworks that may allow to identify regions under major regulatory programs on the chromosome level that could help to understand the general principles of colon cancer.

This paper is organized as follows: In the next section, we describe all methods and data we are using for our analysis. In the 'Results' section, we present our findings and in the section 'Discussion' we interpret our results. The paper finishes with the section 'Conclusions' with a summary.

## Methods

### Gene expression data set

For our study, we use gene expression data from colon cancer tissue samples from the Expression Project for Oncology (expO) (https://expo.intgen.org/geo*/*) microarray database maintained by the International Genomics Consortium (IGC). The data are obtained from the GEO NCBI repository (*GSE2109 *) [16] containing a total of 289 Affymetrix samples in *CEL *format from the platform *hgu133plus2*. The 289 samples correspond to a number of different histologies, as shown in Table 1, and 149 samples are from female and 139 are from male patients.

**Table 1:** Overview of the histologies of the 289 colon cancer samples provided by Expression Project for Oncology (expO).

Histology	Number of Samples
Adenocarcinoma	218
Mucinous Adenocarcinoma	36
Adenocarcinoma arising in a villous adenoma	15
Metastatic Papillary Serous Adenocarcinoma	3
Carcinoma in situ arising in a villous adenoma	2
Metastatic Mucinous Adenocarcinoma	2
Adenocarcinoma In situ	1
Clear cell adenocarcinoma	1
Colloid Carcinoma	1
Medullary Carcinoma	1
Metastatic Adenocarcinoma	1
Metastatic Papillary Serous Carcinoma	1
Metastatic Serous adenocarcinoma (papillary serous)	1
Signet Ring Cell Carcinoma	1
Undifferentiated Carcinoma	1
Missing	4

### Preprocessing and normalization of the data

We normalize the microarray samples for the selected tissue types using RMA and quantile normalization [17] using *log*_2 _expression intensities for each probe set. Because a gene can be represented by more than one probe set, we use the median expression value as summary statistic for different probe sets. Entrez gene ID to Affymetrix probe set annotation is obtained from the *"hgu133plus2.db" *R package. If a probe set is unmapped, we exclude it from our analysis. After these preprocessing steps, we have 19, 738 genes and 289 samples we use for our analysis.

### Inference of the colon cancer gene regulatory network

In recent years many network inference methods have been introduced [18-21]. In this paper, for inferring the colon cancer network from gene expression data, we use the BC3Net algorithm [15], because it has been demonstrated that BC3Net does not only lead to meaningful biological results but it possess also a favorable computational complexity making a large-scale analysis feasible [15,22].

Briefly, BC3Net is a bagging version of C3Net [23,24] that generates from one dataset, *D*, an ensemble of *B *independent bootstrap datasets, ${\left\{D_{k}^{b}\right\}}_{k=1}^{B}$, by sampling from *D *with replacement by using a non-parametric bootstrap with *B *= 100. Then, for each generated data set $D_{k}^{b}$ in the ensemble, a network $G_{k}^{b}$ is inferred by using C3Net [23,24]. From the ensemble of networks ${\left\{G_{k}^{b}\right\}}_{k=1}^{B}$ we construct one aggregate network, $G_{w}^{b}$, which is used to determine the statistical significance of the connection between gene pairs. Then we test the significance of each edge using a binomial test. This results in the final network *GBC*3Net

### Census cancer and colon cancer specific genes

The Cancer Gene Census (CGC) [25] (Version 2011 − 03 − 22, Table 1*full *2011 − 03 − 22) (http://www.sanger.ac.uk/genetics/CGP/Census/) provides information about genes that are frequently observed within tumors of different types of cancer. The CGC list comprises a total of 457 cancer genes, from these 457 genes, 440 are present in the colon cancer gene expression data set.

### CSPNN: Connected shortest path neighbor network

In order to analyze subnetworks of the whole colon cancer gene regulatory network, we extract a *connected shortest path neighbor network *(CSPNN) in the following way. First, we define a set of genes, *L*_1_, e.g., by using cancer genes. Then we determine all shortest paths between these genes using the Dijkstra distance [26]. This results in a second set of genes that contains all genes on these shortest paths, including the genes in *L*_1_, we call *L*_2_. Mapping *L*_2 _onto the network *GBC*3Net gives us a connected subnetwork. To thissubnetwork we add all next neighbors of the genes in *L*_1 _resulting in the CSPNN.

### GPEA: Gene pair enrichment analysis

It has been shown that genes that cluster together in a co-expression network share a common biological function [27]. We extend this analysis to take the connectivity structure of a gene regulatory network into more detailed account. Specifically, for testing the statistical enrichment of GO-terms in the inferred colon cancer network, we are applying a hypergeometric test that is based on 'interactions' (edges). Due to the fact that 'interactions' always involve a 'pair of genes' this test is called **g**ene **p**air **e**nrichment **a**nalysis (GPEA) [15,28]. For our analysis, we obtain information from the Gene Ontology database for entrez IDs of genes from the Bioconductor [29] annotation packages *org.Hs.eg.db *(v2.9.0) and *GO.db *(v2.9.0).

In the following, we briefly describe a GPEA. In this description, we use the terms 'interaction', 'edge' and 'gene pair' synonymously. For p genes there is a total of *N *= *p*(*p *− 1)/2 different gene pairs. If there are *p_GO _*genes for a particular GO-term then the total number of gene pairs for this GO-term is *m_GO _*= *p_GO _*(*p_GO _*− 1)/2. Furthermore, if we suppose that the inferred colon cancer network *GBC*3Net contains *n *interactions, of which *k *interactions are among genes from the given GO-term, then a p-value for the enrichment of gene pairs of this GO-term can be calculated from the following hypergeometric distribution

(1) $$p\left(k\text{|GO - term}\right)=\overset{m_{GO}}{\underset{i=k}{\sum }}P\left(X=i\text{|GO-term}\right)=\overset{m_{GO}}{\underset{i=k}{\sum }}\frac{\left(\begin{array}{c} m_{GO}\\ i \end{array}\right)\left(\begin{array}{c} N-m_{GO}\\ n-i \end{array}\right)}{\left(\begin{array}{c} N\\ n \end{array}\right)}$$

This p-value gives an estimate for the probability to observe *k *or more interactions between genes from the given GO-term.

### Chromosome cooperativity analysis

For analyzing the 'cooperativity' among chromosomes, we define a statistical test that estimates if there are chromosome pairs that contain a statistically significant number of interactions between them [30]. For instance, for chromosome *i *and *j *we calculate the number of interactions, *s_i,j_*, from the colon cancer network *GBC*3Net and apply a statistical hypothesis test to see if this number is larger than expected by chance, i.e., $s_{\text{rand}|i,j}$

We obtain the sampling distribution for the null hypothesis

(2) $$H_{0}:s_{i,j}=s_{\text{rand|}i,j}\;\;\;\;\;\;\;\text{for }i,j\in \left\{1,2,\cdots \;,X,Y\right\}$$

from gene label randomizations in the colon cancer network. For our analysis we used *E *= 100, 000.

For each randomization, *e *∈ *E*, we calculate the number of interactions $s_{i,j}^{e}$ between each chromosome pair (i,j∈{1,2,⋯,22,X,Y} from which we estimate the p-values by

(3) $$p_{i,j}=\frac{\sum_{e=1}^{E}I\left(s_{i,j}^{e}>s_{i,j}\right)}{E}$$

Here, *I*(), is the indicator function that gives a value of '1' if its argument is true and '0' otherwise. We would like to emphasize that by utilizing the connectivity structure of the colon cancer network *GBC*3Net in combination with a gene label resampling will conserve not only the total number of interactions among genes, but also the structural properties of the network. Also the uneven number of genes on the 24 chromosomes is accommodated by our resampling procedure. In total, we perform 300 = (24^2 ^− 24)/2 + 24 tests and adjust for multiple testing by applying a Benjamini & Hochberg [31] correction controlling the FDR for a significance level of α = 0.05. This guarantees a false discovery rate of FDR ≤ α [32].

## Results

### Colon cancer gene regulatory network

Using the gene expression data set from expO and the BC3Nnet algorithm, we infer a colon cancer gene regulatory network (GRN), briefly denoted as *GBC*3Net.This regulatory network consists of 19, 738 genes and contains 135, 194 interactions (edges) among these genes. With the exception of 14 genes the overall colon cancer network is connected. Technically, this means that the giant connected component (GCC) [33] of our colon cancer network has a size of 19, 724 genes. For this network, we find an average shortest path length of 4.52 (measured with the Dijkstra distance [34]) and an edge density of $\in =6.9\cdot 10^{-4}$. The degree distribution of the colon cancer network follows a power law distribution with an exponent of α = 3.22 indicating that the resulting network is *scale-free *[35], as has been previously found for many different types of biological networks [36-38], including GRNs [30,39].

### Functional GPEA of biological processes

We evaluate our colon cancer GRN network based on functional knowledge about genes that are involved in similar biological processes as defined in the Gene Ontology (GO) database [40]. On the assumption that functionally related genes are likely to interact with each other, we sought to identify the functional modules that are most prominently represented in our inferred colon cancer GRN network. For this reason, we perform a GPEA analysis for GO-terms with a term size larger than 2 and less than 1000 genes and a significance level ofα = 0.001 with a Bonferroni multiple testing correction. Furthermore, in order to study the relevance of the identified functional modules for cancer hallmarks, we test for the enrichment of cancer census genes [25].

In total, we test 7, 989 GO-terms from the category Biological Process and find 430 (5.38%) statistically significant terms. The 50 most significant terms of the GPEA analysis are shown in Table 2. The significant GO-terms describe a variety of biological processes such as cell cycle phase (938 edges), translational initiation (155 edges), elongation (156 edges) and termination (130 edges), organelle fission (318 edges), viral transcription (137 edges), cellular respiration (122 edges), type I interferon-mediated signaling pathway (62 edges) and regulation of immune system process (609 edges).

**Table 2:** Biological Process GPEA analysis showing the 50 most significant terms.

GOID	GO-term	#Genes	#Interactions	p-value	GCC	CG
GO:0022403	cell cycle phase	853	938	5.8e-238	349	60/+
GO:0000278	mitotic cell cycle	776	818	7.1e-221	343	54/+
GO:0006414	translational elongation	108	156	3.0e-181	72	1
GO:0006415	translational termination	91	130	9.0e-160	67	1
GO:0006614	SRP-dependent cotranslational protein targeting to membrane	105	136	4.6e-153	67	2
GO:0045047	protein targeting to ER	107	137	2.1e-152	67	2
GO:0072599	establishment of protein localization to endoplasmic reticulum	108	137	2.6e-151	67	2
GO:0006613	cotranslational protein targeting to membrane	107	136	7.4e-151	67	2
GO:0000279	M phase	537	462	4.1e-149	196	33/+
GO:0000087	M phase of mitotic cell cycle	374	321	3.6e-144	159	20/+
GO:0070972	protein localization to endoplasmic reticulum	121	140	2.2e-142	67	2
GO:0000184	nuclear-transcribed mRNA catabolic process, nonsense-mediated decay	118	137	6.0e-141	70	2
GO:0000280	nuclear division	363	305	7.2e-138	155	20/+
GO:0007067	mitosis	363	305	7.2e-138	155	20/+
GO:0006413	translational initiation	153	155	7.4e-134	78	4
GO:0048285	organelle fission	388	318	4.0e-133	161	20/+
GO:0006412	translation	469	355	5.2e-115	183	16
GO:0000956	nuclear-transcribed mRNA catabolic process	171	150	1.1e-113	73	7
GO:0006612	protein targeting to membrane	154	139	7.9e-113	67	4
GO:0019080	viral genome expression	152	137	7.7e-112	70	10/+
GO:0019083	viral transcription	152	137	7.7e-112	70	10/+
GO:0016071	mRNA metabolic process	614	463	4.2e-109	301	21
GO:0006402	mRNA catabolic process	183	152	1.2e-107	73	7
GO:0043624	cellular protein complex disassembly	157	131	5.9e-101	67	2
GO:0043241	protein complex disassembly	162	132	9.1e-99	67	2
GO:0006401	RNA catabolic process	210	157	5.1e-96	74	7
GO:0072594	establishment of protein localization to organelle	212	156	7.8e-94	74	4
GO:0022904	respiratory electron transport chain	111	97	4.0e-90	62	5
GO:0019058	viral infectious cycle	228	158	7.2e-87	81	14/+
GO:0032984	macromolecular complex disassembly	183	133	7.8e-87	67	7
GO:0045333	cellular respiration	163	122	1.3e-86	80	9/+
GO:0006259	DNA metabolic process	880	655	2.9e-85	334	75/+
GO:0051301	cell division	480	310	2.2e-81	126	35/+
GO:0022900	electron transport chain	151	105	2.0e-74	66	5
GO:0006396	RNA processing	656	428	1.1e-73	249	18
GO:0060337	type I interferon-mediated signaling pathway	73	62	3.2e-67	29	5
GO:0071357	cellular response to type I interferon	73	62	3.2e-67	29	5
GO:0034340	response to type I interferon	74	62	1.7e-66	29	5
GO:0002682	regulation of immune system process	893	609	1.2e-63	265	83/+
GO:0051320	S phase	148	89	2.7e-58	40	8
GO:0045087	innate immune response	544	308	1.8e-56	151	25/+
GO:0051325	interphase	405	218	8.8e-56	116	34/+
GO:0022411	cellular component disassembly	295	156	3.7e-55	69	12
GO:0016032	viral reproduction	701	419	1.5e-54	150	46/+
GO:0044764	multi-organism cellular process	703	420	2.5e-54	150	46/+
GO:0022415	viral reproductive process	547	305	4.6e-54	107	44/+
GO:0051329	interphase of mitotic cell cycle	399	210	3.8e-53	114	34/+
GO:0050776	regulation of immune response	564	313	2.2e-52	146	43/+
GO:0030198	extracellular matrix organization	209	110	5.5e-52	54	11/+
GO:0043062	extracellular structure organization	210	110	1.4e-51	54	11/+

Significant enrichment of cancer census genes is indicated by a '+' (column seven). GCC denotes the size of the giant connected component corresponding to the genes of a GO-term; CG number of census cancer genes in the GCC.

From the 457 defined cancer census genes 440 are present in our colon cancer GRN. In Table 2, we show for each GO-term the number of cancer census genes (column seven - CG). For these, we perform a cancer census gene enrichment analysis using a hypergeometric test with a significance level of α = 0.05 and a Benjamini & Hochberg correction. Overall, from the 50 most significant GO-terms in Table 2, we find 23 to be enriched with cancer genes (indicated in Table 2 by "+"). Overall, the 50 most significant GO-terms comprise in total 4, 197 genes, of which 228 are cancer genes (51.81% = 228/440 of all census genes present in the colon cancer network).

In Additional file 1, we show a table with all 458 significant GO-terms.

### Core subnetwork of colon cancer genes

In order to learn about the immediate interactions between well known colon cancer genes, we extract a *connected shortest path neighbor network *(CSPNN - see 'Methods' section) from our colon cancer network in the following way. For the 6 known colon cancer genes *L*_1 _= {*APC, MLH1, TP53, SMAD4, KRAS *and *BRAF*}, we determine all shortest paths between these genes in *GBC*3Net. This results in the gene set *L*_2 _containing all genes on these shortest paths. Mapping *L*_2 _back onto *GBC*3Net gives us a connected subnetwork to which we add the next neighbor genes of *L*_1_. This results in the CSPNN containing in total 107 genes and 184 interactions. Among the 107 genes are 7 known cancer genes (in addition to the 6 colon cancer genes it contains PRDM16 from the cancer census gene list).

**Figure 1:**
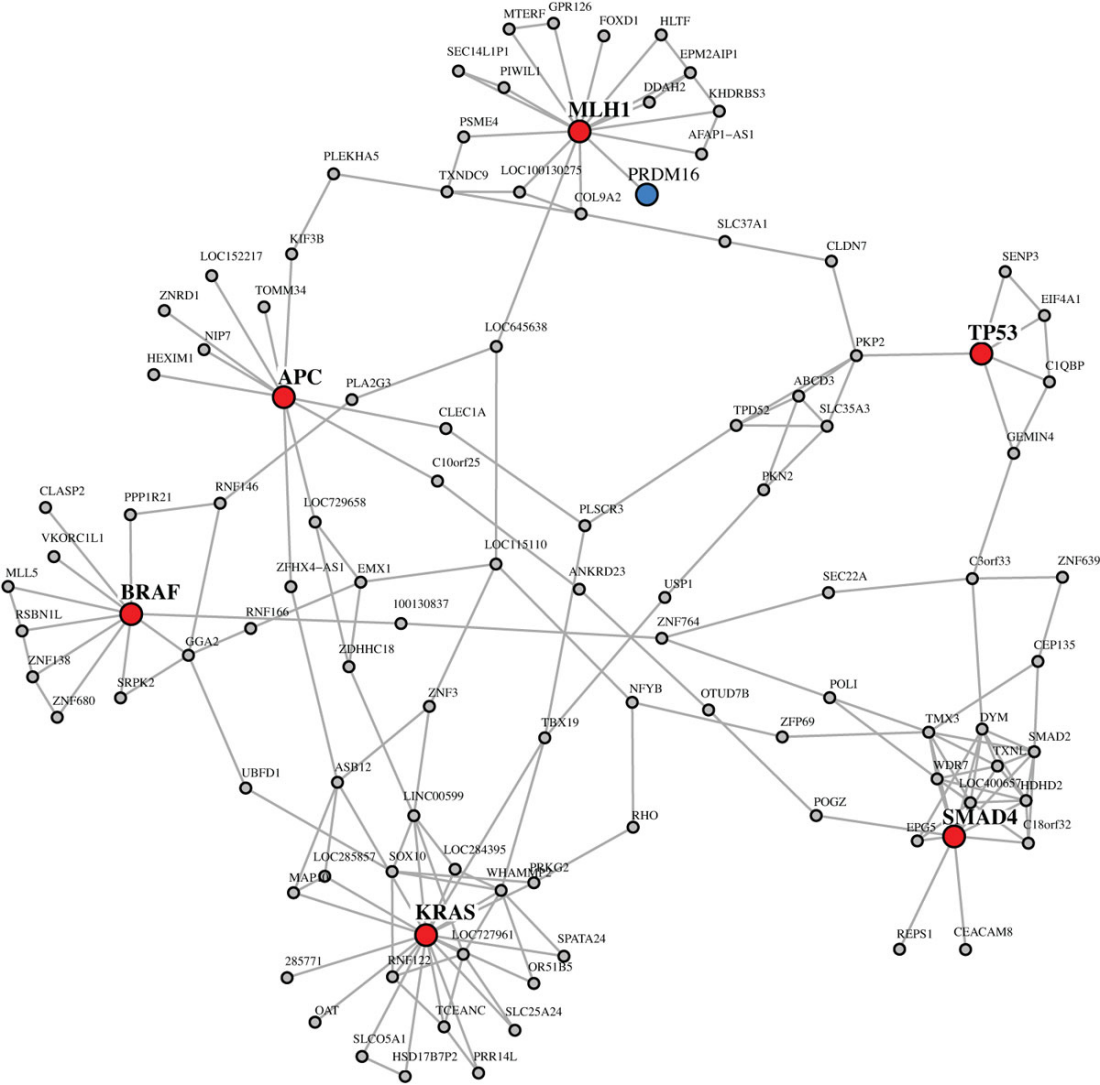
**CSPNN for the 6 colon cancer genes *APC, MLH1, TP53, SMAD4, KRAS *and *BRAF *(red)**. Genes on shortest paths and next neighbor genes are shown in gray besides if they are present in the census cancer gene list (PRDM16 (blue)). In total, this network contains 107 genes, including 7 census cancer genes, and 184 interactions.

Figure 1 shows a graphical visualization of this network. Its average shortest path length is 4.6 and from a functional GPEA, we find as most significant biological process 'macromolecular complex assembly' (GO:0071363), with a nominal p-value of *p_nominal _*= 4.3*e *− 5. It is interesting to observe the interaction between the tumor supressor *APC *and the motor protein *KIF3B. KIF3B *belongs to a microtuble dependent motor protein complex (*KIF3A*-*KIF3B *-*KAP3 *) that is a suggested transport mechanism of the *APC *protein along microtubles [41]. The interaction between the tumor supressor *TP53 *and the SUMO-specific protease *SENP3 *was reported in [42]. *SENP3 *is suggested as a regulator of the *p53-Mdm2 *pathway. We also observe an interaction between *SMAD2 *and *SMAD4. SMAD2 *and *SMAD4 *are both members of the *SMAD *protein complex [43]. Further, *SMAD4 *shows a direct connection to *CEACAM8. CEACAM8 *belongs to the *CEA *gene family and is involved in cell adhesion and migration. The measurement of *CEA *levels in serum is used in the clinic for monitoring the recurrence of colorectal cancer [44].

### Linking interactions in the colon cancer network with their genetic origin

Next, we study the relation between the genetic context and the structural connectivity of our colon cancer network *GBC*3Net in the following way. Interactions between genes on separate or the same chromosome can be seen as *trans-interactions *and *cis-interactions*, analogous to the trans- and cis-regulation of genes [45]. However, we would like to emphasize that there is a crucial difference between both types of connections. For 'regulation', the transcription of a gene is controlled by a cis- or trans-acting transcription factor, whereas an 'interaction' means *any type *of biochemical binding, not limited to transcription regulation, but also including protein-protein interaction, phosphorylation, ubiquitination or others. For our colon cancer network, we find that in total 27, 345(21.01%) interactions are cis-interactions and 102, 806(78.99%) edges correspond to trans-interactions.

In the following, we study three questions that address different chromosomal levels. First, we study the cooperativity of chromosomes in form of the enhancement of their interactions. This identifies pairs of chromosomes that are more cooperative with each other. Second, we study the inferrability of interactions in the colon cancer network with respect to their cis- or trans-acting role. This allows to us to learn about the heterogeneity of these interaction types. Third, we investigate chromosomal neighborhoods with respect to their functional enrichment of GO-terms of the structural connectivity in the colon cancer network.

### Chromosome cooperativity

**Figure 2:**
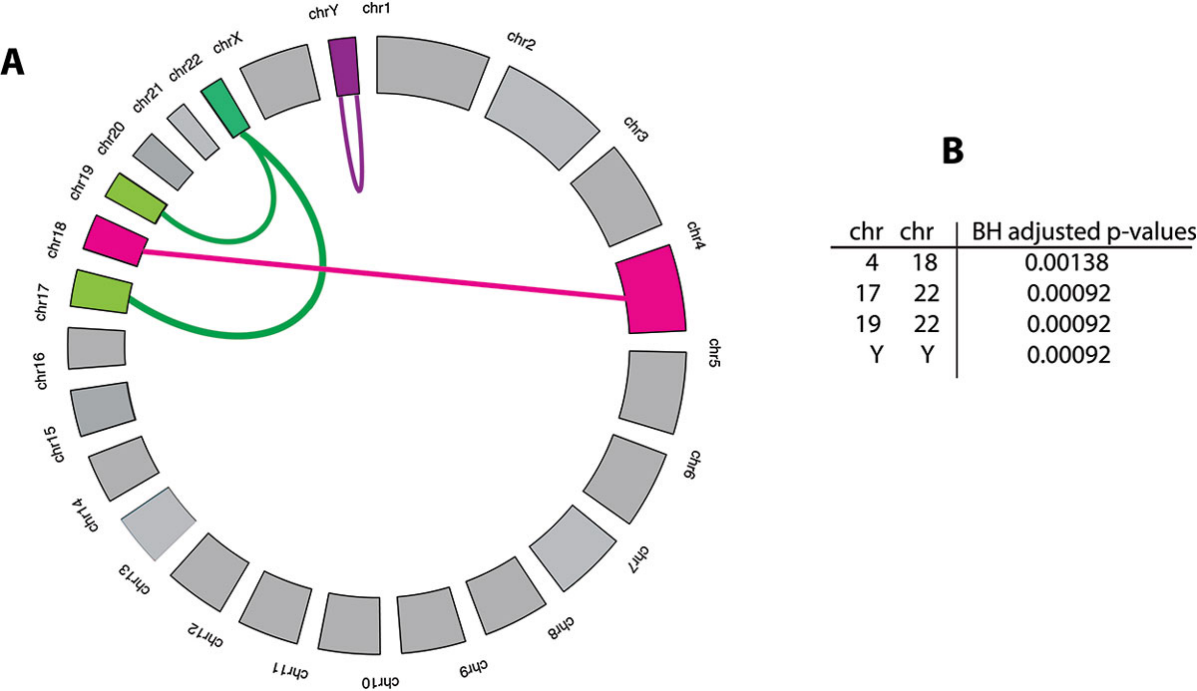
**A: Statistically significant chromosome cooperations are highlighted by a link**. B: The table shows the Benjamini & Hochberg (BH) adjusted p-values for these links.

To enhance insight about the chromosome cooperativity, we conduct a statistical test as described in the Methods section 'Chromosome cooperativity analysis'. As a result, we find that 4 of the 300 chromosome pairs are statistically significant, shown in the table in Figure 2B. It is interesting to note that chromosome 22 is involved in two of these four connections. This is highlighted in Figure 2A by the link color green for Chr 22.

Our analysis also shed light on the *cooperation *of genes as measured by the prevalence of significant interactions between chromosome pairs. From this perspective, visualized in Figure 2A, one sees that only a rather limited number of chromosomes contribute to this *cooperation *on the chromosome level.

#### *Heterogeneity of cis- and trans-interactions*

**Figure 3:**
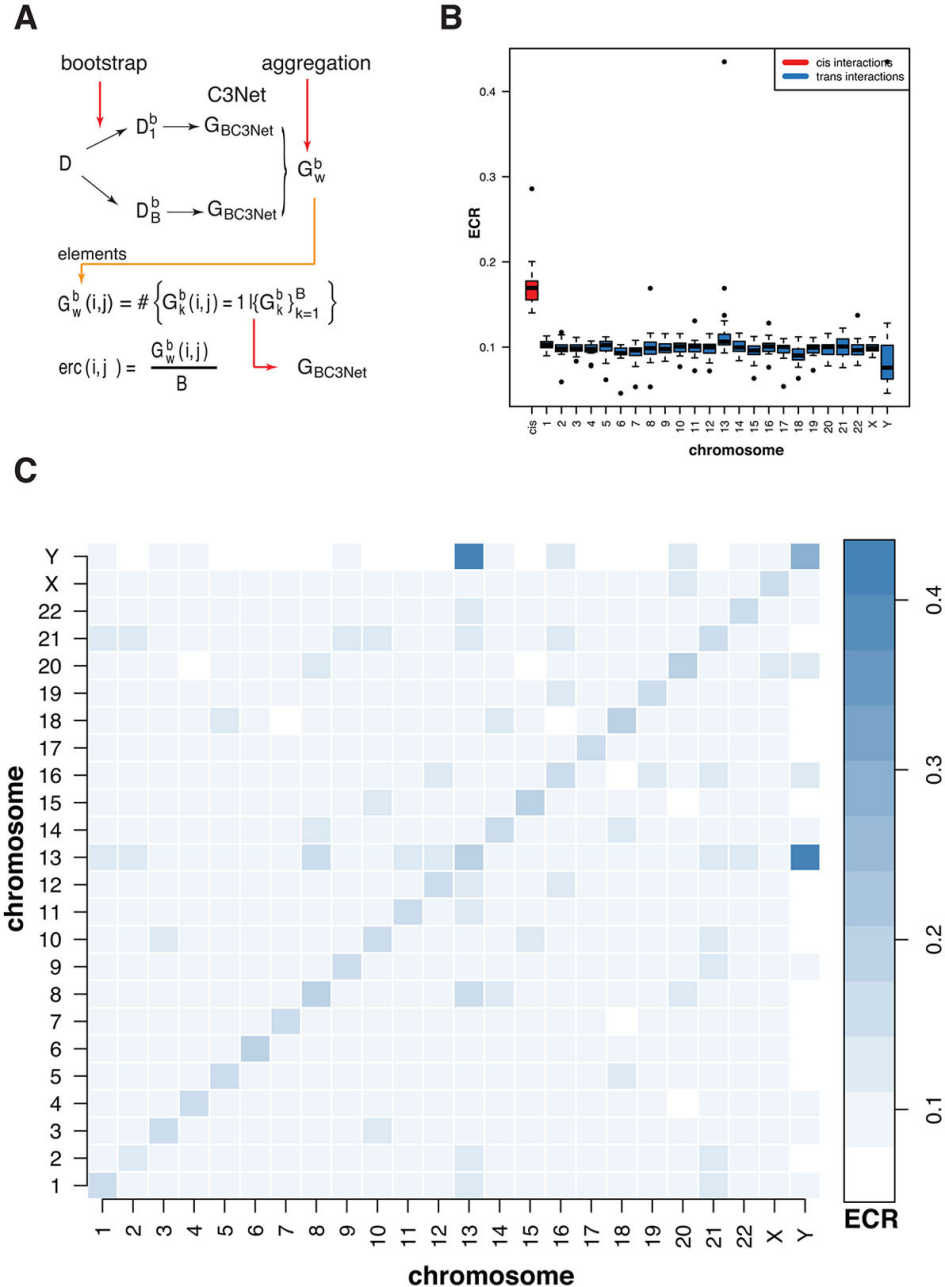
**A: Connection between the ensemble consensus rate and BC3Net**. B: Integrated ensemble consensus rate (ECR) for cis-interactions (red) and trans-interactions (blue). C: Median values of the individual ensemble consensus sets ECS*^mn ^*for m,n∈{1,⋯X,Y}.

To investigate the heterogeneity of cis- and trans-interactions in the colon cancer network, we utilize a measure called the *ensemble consensus rate *(ECR). Specifically, the colon cancer network inferred by BC3Net is aggregated from a bootstrap ensemble of individual networks ${\left\{G_{k}^{b}\right\}}_{k=1}^{B}$; see Figure 3A. This aggregation step is based on the *ensemble consensus rate *(ECR) that measures how often an interaction is observed in the individual networks in the bootstrap ensemble. Formally, the ensemble consensus rate, ecr(*i, j*), is estimated for each potential interaction between gene *i *and gene *j*, as the following probability,

(4) $$\text{ecr}\left(i,j\right)=\text{Pr}\left(\text{finding an interaction between genes i and j in }{\left\{G_{k}^{b}\right\}}_{k=1}^{B}\right).$$

Due to the symmetry of the mutual information values utilized by C3Net, each of the bootstrap ensemble networks in ${\left\{G_{k}^{b}\right\}}_{k=1}^{B}$ is undirected and it holds, ecr(*i, j*) = ecr(*j, i*).

In the following, we want to *zoom-in *potential effects of the chromosomal position of interacting genes on the structure of the colon cancer network. In order to accomplish this, we utilize the ECR from which this network is inferred. Specifically, for each chromosome, we determine the ECR of cis-interactions, between co-located genes on the same chromosome, and trans-interactions, between genes located on different chromosomes. This means, for each pair of chromosomes, m,n∈{1,2,⋯X,Y}, we determine the following set,

(5) $$\text{EC}{\text{S}}^{mn}=\left\{\text{ecr}\left(i,j\right)\text{|gene i is on chromosome m, and gene j is on chromosome n}\right\}.$$

We call the set ECS*^mn ^*the *ensemble consensus set *for chromosome *m *and *n*, because it contains all ECR values of the corresponding interacting genes that are located on chromosome *m *and *n*. As a consequence of symmetry of the ECR also the ensemble consensus sets are symmetric,

(6) $$\text{EC}{\text{S}}^{mn}=\text{EC}{\text{S}}^{nm}.$$

For *m *= *n *these sets correspond to cis-interactions and for *m *≠ *n *to trans-interactions. This means, in total, we have 24 ensemble consensus sets for cis-interactions, $\left\{\text{EC}{\text{S}}^{1,1},\text{EC}{\text{S}}^{2,2},\cdots \text{EC}{\text{S}}^{Y,Y}\right\}$, and 276 ensemble consensus sets for trans-interactions, $\left\{\text{EC}{\text{S}}^{1,2},\text{EC}{\text{S}}^{1,3},\cdots \text{EC}{\text{S}}^{Y,22},\text{EC}{\text{S}}^{Y,X}\right\}$.

The above separation in cis- and trans-interaction types allows a basic understanding of the wiring of the colon cancer network, conditioned on the chromosomes. We start our analysis by presenting results for *integrated *ensemble consensus sets, for a simplified overview. Here by *integrated *we mean an union over chromosomes. For the cis- and trans-interactions that means

(7) $$ECS^{cis}=\underset{m\in \left\{1,\cdots Y\right\}}{⋃}\left\{\text{EC}{\text{S}}^{m,m}\right\}$$

(8) $$ECS^{trans}\left(n\right)=\underset{m\in \left\{1,\cdots Y\right\}}{⋃}\left\{\text{EC}{\text{S}}^{n,m}\right\}\;\;\text{for }n\in \left\{1,\cdots Y\right\}$$

In Figure 3B, we show a boxplot of the distributions of the average ECR rates for the 25 ensemble census sets; *ECS^cis ^*in red and the *ECS^trans^*(*n*) in blue. We observe almost a two-fold higher ECR for cis-interactions (median of means value is 0.1695) compared to trans-interactions (median of means value is 0.0993).

For the distribution of the trans-interactions (blue - Figure 3B) the chromosomes exhibit subtle variations. Chromosome 13 shows the largest and chromosome *Y *shows the smallest median ECR. In order to test, whether this observation is influenced by genes with a large degree, we compared the distribution of the average degree of trans gene pairs between the chromosomes and investigated the location of hub genes. As a result, we found that chromosome 13 has an increased average node degree, compared to all other chromosomes (not shown).

Table 3 shows the 10 major hub genes of the colon cancer network. For each hub gene, we extracted the subnetwork including its direct neighbors. The molecular function of the subnetworks for each hub gene are described by the most significant GO term identified by a Gene Ontology enrichment analysis (*FDR *= 0.1 and a Benjamini & Hochberg correction). The identified terms for the hub gene subnetworks have functional annotations related to cell adhesion and signaling such as synaptic transmission, detection of stimulus, sensory perception and receptor activity (Table 3).

**Table 3:** The 10 major hub genes of the colon cancer network.

entrez	symbol	Description	degree	locus	most significant GO-term
81137	OR7E104P	olfactory receptor	458	chr13q21.31	GO:0007268 synaptic transmission
2623	GATA1	transcription factor	321	chrXp11.23	GO:0007601 visual perception
348808	NPHP3-AS1	antisense RNA	262	chr3q22.1	GO:0050906 detection of stimulus involved in sensory perception
285877	POM121L12	transmembrane protein	247	chr7p12.1	GO:0007606 sensory perception of chemical stimulus
283933	ZNF843	zinc finger protein	231	chr16p11.2	GO:0030534 adult behavior
60506	NYX	extracellular matrix	217	chrXp11.4	GO:0042749 regulation of circadian sleep/wake cycle
387601	SLC22A25	anion transporter	216	chr11q12.3	GO:0048511 rhythmic process
284805	C20orf203	ORF	212	chr20q11.21	GO:0006813 potassium ion transport
6521	SLC4A1	anion transporter	208	chr17q21.31	GO:0072529 pyrimidine-containing compound catabolic process
163778	SPRR4	envelope precursor	207	chr1q21.3	GO:0007608 sensory perception of smell

The hub genes are described by their entrez gene id, gene symbol, short description, node degree, chromosomal location and the most significant GO-term based on a Gene Ontology enrichment analysis based on the direct interactions for each hub gene.

The major hub gene *OR7E104P *is located on chromosome 13 with a degree of 458 (Table 3). The *ECS^trans ^*median of means for chromosome 13 is 0.1108 (Figure 3B) and drops to 0.0953 (not shown) similar to the other chromosomes upon removal of the major hub *OR7E104P*. Hence, the subtle increase of the ECR for chromosome 13 is a result of the largest hub gene of the colon cancer network.

In Figure 3C, we show results for the 300 individual ensemble consensus sets ECS*^mn^*. For reasons of simplicity, we show only the median ensemble consensus rates instead of box plots, to obtain a compressed visualization. Overall, we observe also for the individual ECS higher cis- than trans- consensus rates. Furthermore, chromosome 13 and chromosome Y appear elevated and demeaned (see column colors).

#### *Chromosomal neighborhood-induced GPEA analysis*

**Figure 4:**
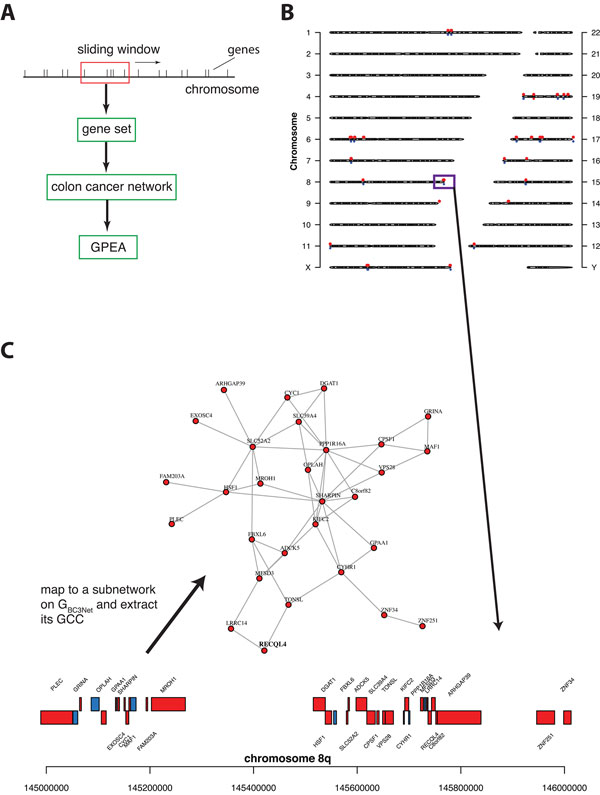
**A: Analysis procedure for a GPEA**. B: Shown are the locations of the largest 146 network components corresponding to gene sets of 1 Mb windows (red dots) along the chromosomes. Blue dots indicate the location of cancer census genes. C: The top ranked largest network component corresponding to the positional gene set on chromosome 8 with 29 genes (red).

Finally, we study the connection between chromosomal neighborhoods and interactions between genes, as given by the colon cancer network. Specifically, we want to identify genomic regions with enriched subnet- works of interacting genes that are adjacent, i.e., co-located, on the chromosomes. This analysis is based on a GPEA where the gene sets are defined from a sliding window along the human chromosome, comprising co-located genes within such a window. See Figure 4A for a schematic visualization and the definition of our gene sets. For our analysis, we use a window length of 1 Mb (mega bases) and slide this window in steps of 500 Kb (Kilo bases) along the chromosomes. That means consecutive windows have an overlap of 500 Kb. We perform a GPEA for a total of 3, 987 chromosome window gene sets, whenever a window contains at least 2 genes that are present in the colon cancer GRN.

From our analysis, we find 260 (6.52%) of the 3, 987 gene sets with a significant enrichment of interactions (α = 0.001 and Bonferroni correction). The 35 most significant genomic regions from this GPEA are shown in Table 4. In this table, each row corresponds to one window gene set and the first column indicates the chromosome, the second the locus and the third the start base pair. Column four and five give the number of genes in the window gene set and the number of edges (interactions) between these genes in the colon cancer network. The p-value in column six corresponds to the result from the GPEA.

**Table 4:** Chromosomal neighborhood-induced GPEA and GO analysis.

chr	locus	start	Size	edges	pvalue	gcc	census	term
chr8	q24.3	145000001	35	52	3.6e-86	29	RECQL4	
chr8	q24.3	145500001	31	37	3.3e-59	24	RECQL4	
chr6	p22.2/p22.1	26000001	45	40	3.5e-52	23		nucleosome assembly (9)
chr6	p22.2	25500001	46	40	2.1e-51	24		nucleosome assembly (9)
chrX	q28	153000001	37	33	1.2e-45	18		
chr19	q13.31	44000001	35	31	1.8e-43	15		regulation of transcription, DNA-
								dependent (15)
chr7	p15.1/.2	27000001	17	21	4.4e-39	12	HOXA9, HOXA11, HOXA13, JAZF1	anterior/posterior pattern specification (9)
chr7	p15.2	26500001	18	21	6e-38	12	HOXA9, HOXA11, HOXA13	anterior/posterior pattern specification (9)
chr8	q24.3	144500001	30	26	1e-37	14		
chr6	p21.1	42500001	32	26	3.3e-36	18		meiosis (3)
chr19	q13.31/q13.32	44500001	28	24	2.7e-35	12	BCL3, CBLC	regulation of transcription, DNA- dependent (12)
chr17	q12/q21.1/.2	37500001	26	22	8.4e-33	13	ERBB2,	
							RARA	
chr17	p13.1	7000001	56	32	3.3e-32	22	TP53	
chrX	q28	153500001	30	22	5.6e-30	14	MTCP1	
chr1	q22	155000001	33	23	6.4e-30	20	MUC1	
chr17	q11.2	26500001	34	23	2.6e-29	17		
chr8	p11.21	42000001	16	16	3.3e-28	11	HOOK3	
chr6	p21.31/.32	32500001	34	22	1.6e-27	7	DAXX	antigen processing and presentation of exogenous peptide antigen via MHC class II (6) proteasomal ubiquitin-dependent protein catabolic process (3)
chr9	q34.3	139500001	53	27	3.4e-26	15		
chrX	p11.23	48500001	28	19	1.5e-25	14	WAS, GATA1, TFE3	
chr17	q21.32	46000001	26	18	7.2e-25	7		embryonic skeletal system development (5)
chr16	p13.3	1500001	47	24	2.5e-24	15	TSC2	protein ubiquitination (4)
chr17	q21.32/.33	46500001	27	18	3e-24	7		embryonic skeletal system development (5)
chr17	p13.1	6500001	51	25	4e-24	20		
chr8	q24.3	144000001	29	18	4e-23	7		heterocycle metabolic process (6)
chr6	p21.33	31000001	54	25	6.8e-23	13		
chr6	p21.32/.33	31500001	54	25	6.8e-23	14		
chr19	q13.43	58000001	40	21	9.2e-23	14		transcription, DNA-dependent (14) regulation of type I interferon- mediated signaling pathway (8) homophilic cell adhesion (8) cellular biosynthetic process (9)
chr9	p21.3	20500001	20	15	1e-22	8	MLLT3	
chr5	q31.3	140000001	52	24	3e-22	8		
chr17	q12	37000001	21	15	4.4e-22	13	LASP1, ERBB2	
chr8	p11.22/.23	37500001	18	14	5.2e-22	8	WHSC1L1, FGFR1	
chr19	q13.43	57500001	35	19	8.4e-22	10		regulation of transcription, DNA-dependent (10)
chr17	q25.3	79500001	46	22	1e-21	20	ASPSCR1	proteasomal ubiquitin-dependent protein catabolic process (3)
chrX	p11.23	48000001	28	16	5.6e-20	10	SSX1, WAS, GATA1, TFE3	

Each row corresponds to a window gene set. These windows are indexed by the chromosome, locus and base start. The number of genes in these windows and the edges between them are given in column four and five. Column six gives the p-value of the GPEA analysis (p-val) and column nine shows the most significant GO term for the genes in the GCC.

Column seven shows the number of genes in the giant connected component (GCC). For these genes we perform a (conventional) Gene Ontology enrichment analysis to characterize the biological function for each window gene set. In column nine, we show the most significant GO term (α = 0.05 and Benjamini & Hochberg FDR correction) as a result from this analysis. Furthermore, we find that 44/260 of the chromosome window subnetworks have a GCC with more than ≥ 10 genes. The genomic locations of these 44 gene sets are visualized in Figure 4B.

The 260 chromosome windows comprise a total of 4,292/18,307 (23.44%) genes with 93/425 (21.88%) cancer census genes. The identified chromosomal locations describe a variety of biological processes that are involved in regulation transcription, nucleosome assembly, cell adhesion, signaling (e.g., TOR signaling, type-I interferon-mediated signaling pathway), cell cycle and antigen processing and presentation (Table 4).

The most significant chromosome window is located on chromosome 8 at 145-146 Mb, which corresponds to the chromosome band 8q24.3. In the literature genomic aberration in the locus 8q24 are frequently observed in colon cancer e.g., [46-48]. Figure 4C shows the corresponding largest connected component on chromosome 8 146-147 Mb with 29 genes including the census cancer gene RECQL4.

## Discussion

In this study, we inferred a colon cancer gene regulatory network and investigated its functional and structural meaning. Overall, we found our colon cancer regulatory network consists of 19, 718 genes interconnected by 135, 194 interactions. Within this network, approximately 5% of the gene ontology (GO) terms we studied were enriched and functional annotations for the 50 most significant GO terms (see Table 2) included 11 that denote gene clusters involved in engagement with cellular and molecular inflammatory mediators or infective agents. Thirteen terms are involved in gene transcription, translation and mRNA degradation implicated in generic signaling processes while 10 had clear association with cell cycle regulation or progression. Five terms had functions in processing of subcellular protein complexes and organelles while a further 7 are associated with protein targeting to membranes or other spatial domains. These 12 terms have key functional annotations required for compartmentalized signaling for control of cytoskeletal dynamics in simultaneous subcellular and cellular processes, including vesicle trafficking, endocytosis, cytokinesis, cell migration and morphogenesis [49,50]. By integration of complex biological information with widely adopted GO terms for major human cancer, this study will enhance the quality and accuracy of functional annotations within emerging GRNs that may be used in predictive cancer science.

The analysis of chromosome cooperativity revealed that there are only very few chromosome paris (1.3% = 4/300) that have an enhanced number of interactions among the genes located on these chromosomes (see Figure 2) and chromosomes 22 is involved in 2 of the 4 significant connections. An increase for trans-interactions between two chromosomes may result from a spatial proximity of the genes in the nucleus leading to an increased co-regulation of gene expression because the spatial organization of chromosomes and the intermingling between chromosomes (*chromosome kissing*) in the nucleus is crucial for the regulation of gene activation, gene silencing and the process of genomic translocations [51,52].

Only by connecting the interaction structure of the colon cancer network with the chromosomal locations of the genes enabled the definition of cis- and trans-interactions. This allowed the analysis of structural properties of the genes in the gene regulatory network with respect to their chromosomal positions. Along these lines, we found that interacting genes that are co-located on the same chromosome were observed to have an almost two-fold higher ensemble consensus rate (ECR) compared to trans-located gene pairs, where the corresponding genes reside on different chromosomes. This result holds for the integrated as well as individual ECRs.

A possible explanation for this observation may be related to the underlying structure of the 'true' gene regulatory network of colon cancer. Specifically, in [53], we found that interactions connecting peripheral genes, i.e., genes with only one or two interactions, are more easy to infer than highly connected genes from the center of a network, e.g., hub genes. Hence, cis-interactions may correspond to interactions between genes in the periphery of the 'true' colon cancer network and trans-interactions connect more densely connected genes. Furthermore, in [53] it was shown that peripheral regions of 'true' gene regulatory networks are enriched for membrane proteins and membrane signaling. Hence, the observed heterogeneity of cis- and trans-interactions in our study may also be related to the known inferential heterogeneity [53] of gene regulatory networks.

From studying the connectivity of chromosomal neighborhoods, we found 260 of such neighborhoods to be statistically significant from a GPEA. Furthermore, we found 44 of these to have ≥ 10 genes. An additional GO enrichment analysis of genes in the GCC of these subnetworks showed that several of these subnetworks are involved in 'DNA dependent transcriptional regulation' (see Table 4). Moreover, 8 significant subnetworks are located on chromosome 17, which had been also identified from our chromosome cooperativity analysis.

A general explanation for the presence of 'DNA dependent transcriptional regulation' among the significant chromosomal neighborhoods is certainly related to the basic coordination of transcription of a cell, because in order to allow the transcription of genes chromatin modifications such as histone acetylations are required to allow the unwinding of DNA and make it accessible for transcriptional activity. Given the complexity of these processes and the energy expended, it is not unsurprising that genes are not randomly distributed on the chromosomes. Instead, it is believed that in a mammalian organism genes involved in regulatory programs can be co-ordinately controlled. For instance, transcriptional analysis of the cell cycle [54] suggests that a quartile of cell cycle regulatory genes are adjacent on the chromosome. Similar results have been found for a cardiac transcriptome [55]. These observations suggest a global regulatory organization of gene expression at the chromosomal level and the location of the chromosome in the nucleus has been shown to exert a major effect on transcriptional activity [56]. Certainly, the simplest form of such co-regulation is that of proximally located genes, typically located within the scale of a few Mb [57].

Co-regulated expression of proximal genes was known for a long time, however, it was assumed that genes are regulated locally, at the level of transcription factors. The first large-scale study of genes expression along chromosomes (Human Transcriptome Map) shed light on the global expression patterns: along human chromosomes, highly expressed genes tend to cluster in large domains, interspersed with domains of weakly expressed genes [58]. Similar spatial patterns of genes expression were found in mouse genome [59] and other model organisms (reviewed in [60]). In the nucleus, clusters of actively transcribed genes tend to co-localize, indicating long-range intrachromosomal interactions [61]. Thus, clustering of highly-expressed genes does not reflect individual gene regulation, but microenviroment of chromosomal domain, defined by chromatin structure and subnuclear localization [62]. Our finding that subnetworks of interacting genes are indeed co-located on the chromosomes indicates that, generally, subnetworks in biological networks have many interesting functional properties, some of them are yet to be discovered.

## Conclusions

An interesting future extension would be a comparative analysis of more than one cancer network to learn about commonalities, and differences, of different cancer types with respect to the hallmarks of cancer. For instance, a comparative analysis of these networks could employ similarity or distance measures based on topological indices [63,64] rather than using classical graph similarity measures [65].

Unfortunately, currently, there are severe practically limitations for such an approach, most notably the lack of a database making such cancer networks available. In this respect, the colon cancer network we inferred in this study can also contribute to such a comparative network analysis, extending its usage significantly beyond a single study.

## Competing interests

The authors declare that they have no competing interests.

## Supplementary Material

Additional file 1 Supplementary file Click here for file

## Declarations

MD thanks the Austrian Science Funds for supporting this work (project P26142).

This article has been published as part of *BMC Bioinformatics *Volume 15 Supplement 6, 2014: Knowledge Discovery and Interactive Data Mining in Bioinformatics. The full contents of the supplement are available online at http://www.biomedcentral.com/bmcbioinformatics/supplements/15/S6.
