# Supplementary material for: Functional and genetic analysis of the colon cancer network
Source: BMC Bioinformatics. 2014 May 16;15(Suppl 6):S6. doi: 10.1186/1471-2105-15-S6-S6 (PMC4158620; doi:10.1186/1471-2105-15-S6-S6)
Supplement: Additional file 1 — Supplementary file [file 1471-2105-15-S6-S6-S1.PDF]

Supplementary: Functional and genetic analysis of the colon cancer network

Frank Emmert-Streib[1,+*], Ricardo de Matos Simoes[1,+], Galina Glazko[2], Simon McDade[3], Benjamin Haibe-Kains[4], Andreas Holzinger[5], Matthias Dehmer[6] and Frederick Charles Campbell[3]

[1] Computational Biology and Machine Learning Laboratory, Center for Cancer Research and Cell Biology, School of Medicine, Dentistry and Biomedical Sciences, Faculty of Medicine, Health and Life Sciences, Queen's University Belfast, 97 Lisburn Road, Belfast BT9 7BL, UK [2] Division of Biomedical Informatics, University of Arkansas for Medical Sciences, Little Rock, AR 72205, USA [3] Center for Cancer Research and Cell Biology, School of Medicine, Dentistry and Biomedical Sciences, Faculty of Medicine, Health and Life Sciences, Queen's University Belfast, 97 Lisburn Road, Belfast BT9 7BL, UK [4] Bioinformatics and Computational Genomics Laboratory, Princess Margaret Cancer Centre, University of Toronto, Department of Medical Biophysics, Canada [5] Institute for Medical Informatics, Statistics and Documentation, Medical University Graz, Auenbruggerplatz 2, 8036 Graz, Austria [6] Institute for Bioinformatics and Translational Research, UMIT, Eduard Wallnoefer Zentrum 1, 6060, Hall in Tyrol, Austria

Email: Frank Emmert-Streib*- v@bio-complexity.com
'+': Both authors contributed equally;

*Corresponding author

Abstract

Functional analysis

Table 1: GPEA Biological Process analysis for the colon cancer GRN. Shown are 430 of 7,989 significant terms for $\alpha = 0.001$ and a Bonferroni multiple testing correction. GCC denotes the size of the *giant connected component* of the GRN for the individual terms. CG shows the corresponding number of cancer census genes for the individual terms. A significant enrichment of census genes for an individual term is denoted by "+".

GOID	Term	#Genes	#Inter.	p-value	GCC	CG
GO:0022403	cell cycle phase	853	938	5.8e-238	349	60/+
GO:0000278	mitotic cell cycle	776	818	7.1e-221	343	54/+
GO:0006414	translational elongation	108	156	3.0e-181	72	1
GO:0006415	translational termination	91	130	9.0e-160	67	1
GO:0006614	SRP-dependent cotranslational protein targeting to membrane	105	136	4.6e-153	67	2
GO:0045047	protein targeting to ER	107	137	2.1e-152	67	2

Continued on next page

Table 1 – *Continued from previous page*

GOID	GO term	#Genes	#Inter.	p-value	GCC	CG
GO:0072599	establishment of protein localization to endoplasmic reticulum	108	137	2.6e-151	67	2
GO:0006613	cotranslational protein targeting to membrane	107	136	7.4e-151	67	2
GO:0000279	M phase	537	462	4.1e-149	196	33/+
GO:0000087	M phase of mitotic cell cycle	374	321	3.6e-144	159	20/+
GO:0070972	protein localization to endoplasmic reticulum	121	140	2.2e-142	67	2
GO:0000184	nuclear-transcribed mRNA catabolic process, nonsense-mediated decay	118	137	6.0e-141	70	2
GO:0000280	nuclear division	363	305	7.2e-138	155	20/+
GO:0007067	mitosis	363	305	7.2e-138	155	20/+
GO:0006413	translational initiation	153	155	7.4e-134	78	4
GO:0048285	organelle fission	388	318	4.0e-133	161	20/+
GO:0006412	translation	469	355	5.2e-115	183	16
GO:0000956	nuclear-transcribed mRNA catabolic process	171	150	1.1e-113	73	7
GO:0006612	protein targeting to membrane	154	139	7.9e-113	67	4
GO:0019080	viral genome expression	152	137	7.7e-112	70	10/+
GO:0019083	viral transcription	152	137	7.7e-112	70	10/+
GO:0016071	mRNA metabolic process	614	463	4.2e-109	301	21
GO:0006402	mRNA catabolic process	183	152	1.2e-107	73	7
GO:0043624	cellular protein complex disassembly	157	131	5.9e-101	67	2
GO:0043241	protein complex disassembly	162	132	9.1e-99	67	2
GO:0006401	RNA catabolic process	210	157	5.1e-96	74	7
GO:0072594	establishment of protein localization to organelle	212	156	7.8e-94	74	4
GO:0022904	respiratory electron transport chain	111	97	4.0e-90	62	5
GO:0019058	viral infectious cycle	228	158	7.2e-87	81	14/+
GO:0032984	macromolecular complex disassembly	183	133	7.8e-87	67	7
GO:0045333	cellular respiration	163	122	1.3e-86	80	9/+
GO:0006259	DNA metabolic process	880	655	2.9e-85	334	75/+
GO:0051301	cell division	480	310	2.2e-81	126	35/+
GO:0022900	electron transport chain	151	105	2.0e-74	66	5
GO:0006396	RNA processing	656	428	1.1e-73	249	18
GO:0060337	type I interferon-mediated signaling pathway	73	62	3.2e-67	29	5
GO:0071357	cellular response to type I interferon	73	62	3.2e-67	29	5
GO:0034340	response to type I interferon	74	62	1.7e-66	29	5
GO:0002682	regulation of immune system process	893	609	1.2e-63	265	83/+
GO:0051320	S phase	148	89	2.7e-58	40	8
GO:0045087	innate immune response	544	308	1.8e-56	151	25/+

Continued on next page

Table 1 – *Continued from previous page*

GOID	GO term	#Genes	#Inter.	p-value	GCC	CG
GO:0051325	interphase	405	218	8.8e-56	116	34/+
GO:0022411	cellular component disassembly	295	156	3.7e-55	69	12
GO:0016032	viral reproduction	701	419	1.5e-54	150	46/+
GO:0044764	multi-organism cellular process	703	420	2.5e-54	150	46/+
GO:0022415	viral reproductive process	547	305	4.6e-54	107	44/+
GO:0051329	interphase of mitotic cell cycle	399	210	3.8e-53	114	34/+
GO:0050776	regulation of immune response	564	313	2.2e-52	146	43/+
GO:0030198	extracellular matrix organization	209	110	5.5e-52	54	11/+
GO:0043062	extracellular structure organization	210	110	1.4e-51	54	11/+
GO:0044265	cellular macromolecule catabolic process	718	421	3.4e-50	103	32/+
GO:0000084	S phase of mitotic cell cycle	140	77	4.0e-49	34	8
GO:0010564	regulation of cell cycle process	440	226	1.2e-48	105	45/+
GO:0001775	cell activation	763	452	2.0e-48	177	74/+
GO:0006260	DNA replication	278	136	6.0e-47	68	22/+
GO:0055114	oxidation-reduction process	565	299	7.8e-46	148	18
GO:0045321	leukocyte activation	556	293	7.9e-46	139	63/+
GO:0015980	energy derivation by oxidation of organic compounds	333	160	1.0e-44	95	16/+
GO:0006974	response to DNA damage stimulus	620	334	1.1e-44	160	61/+
GO:0046649	lymphocyte activation	471	235	8.1e-44	120	61/+
GO:0006091	generation of precursor metabolites and energy	456	226	8.8e-44	123	20/+
GO:0002684	positive regulation of immune system process	558	287	1.2e-42	145	41/+
GO:0006261	DNA-dependent DNA replication	100	55	4.9e-42	34	6
GO:0006397	mRNA processing	409	194	4.2e-41	100	14
GO:0006695	cholesterol biosynthetic process	44	32	1.4e-38	14	1
GO:0006281	DNA repair	399	183	3.7e-38	90	42/+
GO:0000075	cell cycle checkpoint	249	110	6.6e-38	55	28/+
GO:0042110	T cell activation	341	152	1.8e-37	81	47/+
GO:0000236	mitotic prometaphase	86	46	3.7e-37	33	3
GO:0007017	microtubule-based process	436	201	4.4e-37	78	24/+
GO:0006271	DNA strand elongation involved in DNA replication	34	27	2.4e-36	18	0
GO:0007059	chromosome segregation	147	66	2.9e-35	43	12/+
GO:0071156	regulation of cell cycle arrest	272	116	4.1e-35	58	30/+
GO:0016126	sterol biosynthetic process	50	32	5.0e-35	14	1
GO:0022616	DNA strand elongation	37	27	2.5e-34	18	0
GO:0006954	inflammatory response	493	227	4.0e-34	122	18
GO:0000226	microtubule cytoskeleton organization	287	121	4.7e-34	63	21/+
GO:0042590	antigen processing and presentation of exogenous peptide antigen via MHC class I	79	41	7.9e-34	18	0

Continued on next page

Table 1 – *Continued from previous page*

GOID	GO term	#Genes	#Inter.	p-value	GCC	CG
GO:0051276	chromosome organization	707	366	1.0e-33	125	70/+
GO:0008380	RNA splicing	330	140	1.3e-33	61	11
GO:0022613	ribonucleoprotein complex biogenesis	235	98	1.5e-33	28	8
GO:0022610	biological adhesion	965	575	1.5e-33	245	41/+
GO:0033365	protein localization to organelle	516	239	1.9e-33	102	38/+
GO:0034097	response to cytokine stimulus	502	230	3.8e-33	76	32/+
GO:0002474	antigen processing and presentation of peptide antigen via MHC class I	99	47	5.6e-33	19	0
GO:0051249	regulation of lymphocyte activation	296	123	8.5e-33	70	34/+
GO:0007155	cell adhesion	963	570	1.0e-32	240	41/+
GO:0002478	antigen processing and presentation of exogenous peptide antigen	168	70	6.1e-32	28	2
GO:0002479	antigen processing and presentation of exogenous peptide antigen via MHC class I, TAP-dependent	75	38	1.2e-31	17	0
GO:0019221	cytokine-mediated signaling pathway	338	140	1.7e-31	59	26/+
GO:0019884	antigen processing and presentation of exogenous antigen	170	70	2.5e-31	28	2
GO:0006605	protein targeting	484	215	3.5e-31	79	32/+
GO:0048002	antigen processing and presentation of peptide antigen	185	75	6.6e-31	29	2
GO:0044703	multi-organism reproductive process	735	377	1.0e-30	115	49/+
GO:0002696	positive regulation of leukocyte activation	231	92	1.9e-30	58	24/+
GO:0002694	regulation of leukocyte activation	337	137	4.3e-30	80	35/+
GO:0051726	regulation of cell cycle	743	380	7.8e-30	148	78/+
GO:0050867	positive regulation of cell activation	241	95	9.1e-30	58	25/+
GO:0051607	defense response to virus	189	75	9.9e-30	34	7
GO:0009057	macromolecule catabolic process	903	506	1.1e-29	248	41/+
GO:0071345	cellular response to cytokine stimulus	417	175	1.7e-29	69	27/+
GO:0050863	regulation of T cell activation	228	88	1.8e-28	52	27/+
GO:0019882	antigen processing and presentation	215	83	1.9e-28	31	2
GO:0050865	regulation of cell activation	362	145	3.9e-28	81	38/+
GO:0001568	blood vessel development	498	214	1.7e-27	117	37/+
GO:0006886	intracellular protein transport	712	349	3.1e-27	86	43/+
GO:0072358	cardiovascular system development	769	389	7.6e-27	193	58/+

Continued on next page

Table 1 – *Continued from previous page*

GOID	GO term	#Genes	#Inter.	p-value	GCC	CG
GO:0072359	circulatory system development	769	389	7.6e-27	193	58/+
GO:0050870	positive regulation of T cell activation	170	64	2.6e-26	45	20/+
GO:0001944	vasculature development	523	225	3.0e-26	123	38/+
GO:0007051	spindle organization	83	36	5.0e-26	25	6/+
GO:0009615	response to virus	268	100	5.2e-26	42	12
GO:0000216	M/G1 transition of mitotic cell cycle	80	35	6.7e-26	9	0
GO:0002480	antigen processing and presentation of exogenous peptide antigen via MHC class I, TAP-independent	9	12	1.2e-25	8	0
GO:0034660	ncRNA metabolic process	291	108	3.5e-25	63	6
GO:0031145	anaphase-promoting complex-dependent proteasomal ubiquitin-dependent protein catabolic process	83	35	7.7e-25	11	2
GO:0051251	positive regulation of lymphocyte activation	213	77	1.0e-24	53	24/+
GO:0042254	ribosome biogenesis	152	56	1.2e-24	23	4
GO:0051707	response to other organism	586	257	2.4e-24	111	23/+
GO:0034341	response to interferon-gamma	112	43	3.5e-24	28	9/+
GO:0002252	immune effector process	475	193	3.7e-24	83	29/+
GO:0042060	wound healing	610	270	9.6e-24	78	35/+
GO:0070727	cellular macromolecule localization	975	541	2.3e-23	232	55/+
GO:0034613	cellular protein localization	971	536	5.1e-23	232	55/+
GO:0001501	skeletal system development	389	147	5.6e-23	38	35/+
GO:0007050	cell cycle arrest	377	141	8.6e-23	75	41/+
GO:0009607	response to biotic stimulus	614	268	2.6e-22	115	25/+
GO:0000375	RNA splicing, via transesterification reactions	216	74	6.1e-22	40	8
GO:0000377	RNA splicing, via transesterification reactions with bulged adenosine as nucleophile	211	72	9.0e-22	40	8
GO:0000398	mRNA splicing, via spliceosome	211	72	9.0e-22	40	8
GO:0048610	cellular process involved in reproduction	552	228	2.4e-21	78	49/+
GO:0048706	embryonic skeletal system development	114	40	1.2e-20	7	16/+
GO:0030199	collagen fibril organization	37	19	1.4e-20	14	2
GO:0000082	G1/S transition of mitotic cell cycle	199	66	1.8e-20	26	15/+
GO:0016482	cytoplasmic transport	726	335	1.8e-20	88	43/+
GO:0071103	DNA conformation change	190	63	1.9e-20	34	15/+
GO:0050778	positive regulation of immune response	380	137	2.3e-20	45	27/+
GO:0009887	organ morphogenesis	802	388	3.9e-20	81	77/+

Continued on next page

Table 1 – *Continued from previous page*

GOID	GO term	#Genes	#Inter.	p-value	GCC	CG
GO:0045786	negative regulation of cell cycle	451	170	5.3e-20	82	49/+
GO:0031347	regulation of defense response	432	160	9.3e-20	33	24/+
GO:0050878	regulation of body fluid levels	595	248	1.1e-19	37	32/+
GO:0002764	immune response-regulating signaling pathway	252	83	1.6e-19	15	26/+
GO:0002768	immune response-regulating cell surface receptor signaling pathway	143	47	2.1e-19	12	16/+
GO:0051443	positive regulation of ubiquitin-protein ligase activity	82	30	2.2e-19	8	1
GO:0071346	cellular response to interferon-gamma	94	33	3.5e-19	24	9/+
GO:0007599	hemostasis	503	194	5.7e-19	28	29/+
GO:0034655	nucleobase-containing compound catabolic process	775	363	8.0e-19	96	40/+
GO:0050817	coagulation	502	193	8.3e-19	28	28/+
GO:0007596	blood coagulation	499	191	1.2e-18	28	28/+
GO:0048704	embryonic skeletal system morphogenesis	85	30	1.6e-18	6	11/+
GO:0008203	cholesterol metabolic process	115	38	1.7e-18	16	3
GO:0007010	cytoskeleton organization	809	386	2.0e-18	73	49/+
GO:0051351	positive regulation of ligase activity	86	30	3.1e-18	8	1
GO:0060333	interferon-gamma-mediated signaling pathway	75	27	5.0e-18	13	9/+
GO:0048514	blood vessel morphogenesis	438	158	7.5e-18	43	31/+
GO:0035637	multicellular organismal signaling	767	353	9.1e-18	216	22
GO:0050900	leukocyte migration	258	82	1.0e-17	41	16/+
GO:0051438	regulation of ubiquitin-protein ligase activity	96	32	1.3e-17	9	3
GO:0050851	antigen receptor-mediated signaling pathway	123	39	2.0e-17	12	15/+
GO:0045088	regulation of innate immune response	226	70	3.3e-17	15	17/+
GO:0006096	glycolysis	62	23	4.1e-17	11	1
GO:0016125	sterol metabolic process	121	38	5.1e-17	16	3
GO:0002429	immune response-activating cell surface receptor signaling pathway	132	41	5.2e-17	12	15/+
GO:0007346	regulation of mitotic cell cycle	319	103	1.1e-16	48	31/+
GO:0007268	synaptic transmission	655	274	1.3e-16	106	14
GO:0051340	regulation of ligase activity	100	32	1.4e-16	9	3
GO:0016072	rRNA metabolic process	112	35	1.8e-16	23	1
GO:0046700	heterocycle catabolic process	818	384	1.9e-16	96	40/+
GO:0048705	skeletal system morphogenesis	183	55	2.0e-16	8	17/+
GO:0002757	immune response-activating signal transduction	239	73	2.2e-16	14	25/+

Continued on next page

Table 1 – *Continued from previous page*

GOID	GO term	#Genes	#Inter.	p-value	GCC	CG
GO:0006694	steroid biosynthetic process	139	42	3.0e-16	14	3
GO:0001525	angiogenesis	362	119	5.6e-16	15	24/+
GO:0019439	aromatic compound catabolic process	820	383	6.8e-16	99	40/+
GO:0015985	energy coupled proton transport, down electrochemical gradient	21	12	9.8e-16	9	0
GO:0015986	ATP synthesis coupled proton transport	21	12	9.8e-16	9	0
GO:0044270	cellular nitrogen compound catabolic process	818	380	1.5e-15	96	40/+
GO:0046165	alcohol biosynthetic process	132	39	2.4e-15	15	2
GO:0016477	cell migration	851	403	3.0e-15	185	43/+
GO:0006364	rRNA processing	106	32	4.0e-15	18	1
GO:0007093	mitotic cell cycle checkpoint	141	41	5.2e-15	15	15/+
GO:0034470	ncRNA processing	204	59	8.0e-15	35	3
GO:0031570	DNA integrity checkpoint	142	41	8.5e-15	8	16/+
GO:1901361	organic cyclic compound catabolic process	854	403	8.7e-15	99	40/+
GO:0019226	transmission of nerve impulse	745	325	1.0e-14	202	22
GO:0006323	DNA packaging	154	44	1.4e-14	22	9/+
GO:0051436	negative regulation of ubiquitin-protein ligase activity involved in mitotic cell cycle	67	22	1.8e-14	8	1
GO:0016192	vesicle-mediated transport	902	438	1.9e-14	129	31/+
GO:0008202	steroid metabolic process	276	82	2.6e-14	16	5
GO:0051352	negative regulation of ligase activity	72	23	2.8e-14	8	2
GO:0051437	positive regulation of ubiquitin-protein ligase activity involved in mitotic cell cycle	72	23	2.8e-14	8	1
GO:0051444	negative regulation of ubiquitin-protein ligase activity	72	23	2.8e-14	8	2
GO:0009952	anterior/posterior pattern specification	211	60	4.1e-14	9	21/+
GO:0051439	regulation of ubiquitin-protein ligase activity involved in mitotic cell cycle	78	24	7.3e-14	8	2
GO:0000904	cell morphogenesis involved in differentiation	701	292	8.9e-14	79	56/+
GO:0043161	proteasomal ubiquitin-dependent protein catabolic process	226	64	1.1e-13	13	7
GO:0006521	regulation of cellular amino acid metabolic process	58	19	2.7e-13	8	2
GO:0006007	glucose catabolic process	76	23	2.8e-13	11	1
GO:0006310	DNA recombination	216	60	3.4e-13	29	23/+
GO:0010498	proteasomal protein catabolic process	236	66	5.0e-13	13	7

Continued on next page

Table 1 – *Continued from previous page*

GOID	GO term	#Genes	#Inter.	p-value	GCC	CG
GO:0002253	activation of immune response	312	92	7.2e-13	14	25/+
GO:0006812	cation transport	647	254	1.1e-12	142	13
GO:0080134	regulation of response to stress	741	313	1.3e-12	46	51/+
GO:0006977	DNA damage response, signal transduction by p53 class mediator resulting in cell cycle arrest	66	20	2.1e-12	8	5
GO:0072413	signal transduction involved in mitotic cell cycle checkpoint	66	20	2.1e-12	8	5
GO:0072431	signal transduction involved in mitotic cell cycle G1/S transition DNA damage checkpoint	66	20	2.1e-12	8	5
GO:0072474	signal transduction involved in mitotic cell cycle G1/S checkpoint	66	20	2.1e-12	8	5
GO:1901700	response to oxygen-containing compound	762	326	2.2e-12	140	39/+
GO:0006270	DNA replication initiation	29	12	2.8e-12	7	1
GO:0070647	protein modification by small protein conjugation or removal	645	251	3.1e-12	61	33/+
GO:0060338	regulation of type I interferon-mediated signaling pathway	34	13	3.7e-12	8	4/+
GO:0072401	signal transduction involved in DNA integrity checkpoint	67	20	3.7e-12	8	6/+
GO:0072404	signal transduction involved in G1/S transition checkpoint	67	20	3.7e-12	8	5
GO:0072422	signal transduction involved in DNA damage checkpoint	67	20	3.7e-12	8	6/+
GO:0000819	sister chromatid segregation	53	17	3.9e-12	6	1
GO:0072395	signal transduction involved in cell cycle checkpoint	68	20	6.5e-12	8	6/+
GO:0031571	mitotic cell cycle G1/S transition DNA damage checkpoint	73	21	7.8e-12	8	6/+
GO:0050852	T cell receptor signaling pathway	98	26	2.4e-11	9	11/+
GO:0071158	positive regulation of cell cycle arrest	80	22	2.6e-11	9	8/+
GO:0019320	hexose catabolic process	90	24	3.8e-11	11	1
GO:0071779	G1/S transition checkpoint	86	23	5.1e-11	8	10/+
GO:0031398	positive regulation of protein ubiquitination	134	34	5.2e-11	8	6
GO:1901617	organic hydroxy compound biosynthetic process	158	40	5.4e-11	15	3
GO:0048562	embryonic organ morphogenesis	223	58	6.7e-11	7	21/+
GO:0016567	protein ubiquitination	545	190	6.8e-11	31	27/+
GO:0031575	mitotic cell cycle G1/S transition checkpoint	82	22	7.1e-11	8	9/+
GO:0006119	oxidative phosphorylation	73	20	9.1e-11	8	3
GO:0071294	cellular response to zinc ion	11	7	1.2e-10	7	0
GO:0048870	cell motility	925	435	1.3e-10	212	48/+

Continued on next page

Table 1 – *Continued from previous page*

GOID	GO term	#Genes	#Inter.	p-value	GCC	CG
GO:0051674	localization of cell	925	435	1.3e-10	212	48/+
GO:0003002	regionalization	313	87	1.4e-10	10	31/+
GO:0032446	protein modification by small protein conjugation	578	206	1.7e-10	33	29/+
GO:0042776	mitochondrial ATP synthesis coupled proton transport	16	8	3.4e-10	5	0
GO:0006635	fatty acid beta-oxidation	56	16	3.8e-10	9	2
GO:0031396	regulation of protein ubiquitination	182	45	3.8e-10	9	12/+
GO:0046365	monosaccharide catabolic process	95	24	3.9e-10	11	1
GO:0000209	protein polyubiquitination	167	41	4.0e-10	8	5
GO:0006312	mitotic recombination	36	12	4.9e-10	10	2
GO:0009617	response to bacterium	344	97	5.3e-10	29	12
GO:0006935	chemotaxis	600	216	5.4e-10	30	41/+
GO:0042330	taxis	600	216	5.4e-10	30	41/+
GO:0051094	positive regulation of developmental process	699	274	5.5e-10	68	69/+
GO:0019752	carboxylic acid metabolic process	882	398	7.5e-10	214	21
GO:0000902	cell morphogenesis	949	449	8.4e-10	110	64/+
GO:0031397	negative regulation of protein ubiquitination	97	24	9.4e-10	8	4
GO:0016054	organic acid catabolic process	201	49	1.9e-09	18	2
GO:0046395	carboxylic acid catabolic process	201	49	1.9e-09	18	2
GO:0016337	cell-cell adhesion	409	121	2.4e-09	19	16
GO:2000045	regulation of G1/S transition of mitotic cell cycle	104	25	2.5e-09	8	10/+
GO:0033238	regulation of cellular amine metabolic process	75	19	2.6e-09	8	3
GO:0032496	response to lipopolysaccharide	202	49	2.6e-09	11	7
GO:0002237	response to molecule of bacterial origin	213	52	2.9e-09	11	9
GO:0048568	embryonic organ development	344	95	3.0e-09	10	41/+
GO:0000070	mitotic sister chromatid segregation	50	14	4.0e-09	6	1
GO:0006457	protein folding	221	54	4.2e-09	21	3
GO:0030182	neuron differentiation	986	474	4.4e-09	92	72/+
GO:2000602	regulation of interphase of mitotic cell cycle	158	37	5.1e-09	8	18/+
GO:0019886	antigen processing and presentation of exogenous peptide antigen via MHC class II	92	22	6.6e-09	9	2
GO:0044255	cellular lipid metabolic process	840	361	1.0e-08	95	26
GO:0030330	DNA damage response, signal transduction by p53 class mediator	108	25	1.3e-08	8	13/+
GO:0007052	mitotic spindle organization	36	11	1.4e-08	7	2

Continued on next page

Table 1 – *Continued from previous page*

GOID	GO term	#Genes	#Inter.	p-value	GCC	CG
GO:0051603	proteolysis involved in cellular protein catabolic process	429	127	1.4e-08	13	13
GO:0090068	positive regulation of cell cycle process	165	38	1.5e-08	9	15/+
GO:0002521	leukocyte differentiation	333	89	1.6e-08	30	60/+
GO:0043632	modification-dependent macromolecule catabolic process	411	119	1.9e-08	13	13
GO:0046903	secretion	796	329	1.9e-08	50	24
GO:0002495	antigen processing and presentation of peptide antigen via MHC class II	95	22	2.3e-08	9	2
GO:0007389	pattern specification process	433	128	2.3e-08	14	39/+
GO:0030030	cell projection organization	938	431	2.6e-08	111	61/+
GO:0033993	response to lipid	539	177	2.7e-08	24	35/+
GO:0015992	proton transport	70	17	2.9e-08	10	0
GO:0006754	ATP biosynthetic process	54	14	3.1e-08	10	1
GO:0002504	antigen processing and presentation of peptide or polysaccharide antigen via MHC class II	96	22	3.4e-08	9	2
GO:0044257	cellular protein catabolic process	444	132	3.8e-08	16	14
GO:0045597	positive regulation of cell differentiation	502	158	4.8e-08	20	57/+
GO:0050801	ion homeostasis	684	255	6.2e-08	54	26/+
GO:0010043	response to zinc ion	33	10	6.4e-08	7	1
GO:0006818	hydrogen transport	72	17	6.9e-08	10	0
GO:0009062	fatty acid catabolic process	72	17	6.9e-08	10	2
GO:0031295	T cell costimulation	72	17	6.9e-08	7	9/+
GO:0072331	signal transduction by p53 class mediator	131	29	7.0e-08	10	16/+
GO:0006511	ubiquitin-dependent protein catabolic process	402	113	1.0e-07	13	13
GO:0031294	lymphocyte costimulation	73	17	1.1e-07	7	9/+
GO:0019395	fatty acid oxidation	89	20	1.2e-07	10	4
GO:0006936	muscle contraction	255	61	1.2e-07	28	5
GO:0019319	hexose biosynthetic process	68	16	1.3e-07	8	2
GO:0006302	double-strand break repair	115	25	1.8e-07	12	14/+
GO:0008610	lipid biosynthetic process	566	187	2.1e-07	38	18
GO:0032101	regulation of response to external stimulus	382	104	2.3e-07	9	21/+
GO:0007156	homophilic cell adhesion	139	30	2.4e-07	8	3
GO:0006094	gluconeogenesis	64	15	2.4e-07	8	2
GO:0060759	regulation of response to cytokine stimulus	91	20	2.5e-07	8	8/+
GO:0032201	telomere maintenance via semi-conservative replication	24	8	2.7e-07	7	0
GO:0019941	modification-dependent protein catabolic process	408	114	2.8e-07	13	13
GO:0000077	DNA damage checkpoint	135	29	2.8e-07	8	16/+

Continued on next page

Table 1 – *Continued from previous page*

GOID	GO term	#Genes	#Inter.	p-value	GCC	CG
GO:0008284	positive regulation of cell proliferation	668	242	3.2e-07	41	60/+
GO:0043436	oxoacid metabolic process	991	465	3.6e-07	234	26
GO:0034440	lipid oxidation	92	20	3.7e-07	10	4
GO:0030334	regulation of cell migration	398	109	5.6e-07	17	26/+
GO:0044283	small molecule biosynthetic process	456	133	6.0e-07	27	9
GO:0032940	secretion by cell	698	258	7.0e-07	41	21
GO:0042770	signal transduction in response to DNA damage	119	25	7.4e-07	8	15/+
GO:0003012	muscle system process	287	69	7.5e-07	29	6
GO:0055085	transmembrane transport	692	254	8.3e-07	71	16
GO:0007416	synapse assembly	73	16	1.0e-06	5	2
GO:0046364	monosaccharide biosynthetic process	73	16	1.0e-06	8	2
GO:0000722	telomere maintenance via recombination	26	8	1.0e-06	7	0
GO:0045071	negative regulation of viral genome replication	32	9	1.0e-06	6	1
GO:0044242	cellular lipid catabolic process	144	30	1.3e-06	11	2
GO:0051701	interaction with host	411	113	1.3e-06	23	34/+
GO:0032787	monocarboxylic acid metabolic process	433	122	1.3e-06	25	12
GO:2000145	regulation of cell motility	421	117	1.3e-06	17	31/+
GO:0030029	actin filament-based process	469	137	1.7e-06	25	28/+
GO:0010833	telomere maintenance via telomere lengthening	39	10	1.7e-06	7	1
GO:0048525	negative regulation of viral reproduction	33	9	1.8e-06	6	1
GO:0001816	cytokine production	430	120	2.1e-06	49	29/+
GO:0051247	positive regulation of protein metabolic process	899	389	2.2e-06	90	66/+
GO:0065004	protein-DNA complex assembly	137	28	2.5e-06	8	6
GO:0006631	fatty acid metabolic process	307	74	2.7e-06	20	10
GO:0006334	nucleosome assembly	108	22	2.8e-06	8	5
GO:0006839	mitochondrial transport	123	25	2.8e-06	7	3
GO:0072329	monocarboxylic acid catabolic process	87	18	3.0e-06	10	2
GO:0006325	chromatin organization	536	167	3.0e-06	24	58/+
GO:0044282	small molecule catabolic process	260	59	3.6e-06	22	4
GO:0044712	single-organism catabolic process	260	59	3.6e-06	22	4
GO:0007167	enzyme linked receptor protein signaling pathway	886	378	3.7e-06	62	76/+
GO:0009206	purine ribonucleoside triphosphate biosynthetic process	65	14	3.9e-06	10	1
GO:0044711	single-organism biosynthetic process	468	135	4.3e-06	27	9

Continued on next page

Table 1 – *Continued from previous page*

GOID	GO term	#Genes	#Inter.	p-value	GCC	CG
GO:0044057	regulation of system process	452	128	4.5e-06	23	19/+
GO:0034330	cell junction organization	199	42	4.7e-06	6	11/+
GO:0043009	chordate embryonic development	548	172	4.8e-06	16	66/+
GO:0016568	chromatin modification	438	122	4.8e-06	19	55/+
GO:0006006	glucose metabolic process	211	45	5.2e-06	14	6
GO:0002526	acute inflammatory response	110	22	5.5e-06	6	5
GO:0009145	purine nucleoside triphosphate biosynthetic process	66	14	5.8e-06	10	1
GO:0006805	xenobiotic metabolic process	149	30	6.0e-06	6	1
GO:0070085	glycosylation	262	59	6.3e-06	7	6
GO:0009060	aerobic respiration	48	11	6.4e-06	10	6/+
GO:0048598	embryonic morphogenesis	501	149	6.7e-06	13	48/+
GO:0007507	heart development	387	101	7.5e-06	32	38/+
GO:0019048	virus-host interaction	371	95	7.5e-06	13	34/+
GO:0072376	protein activation cascade	84	17	7.5e-06	6	0
GO:0071466	cellular response to xenobiotic stimulus	150	30	8.2e-06	6	1
GO:0050727	regulation of inflammatory response	193	40	8.4e-06	8	10/+
GO:0044403	symbiosis, encompassing mutualism through parasitism	445	124	8.5e-06	25	37/+
GO:0044419	interspecies interaction between organisms	445	124	8.5e-06	25	37/+
GO:0009792	embryo development ending in birth or egg hatching	555	174	1.0e-05	16	66/+
GO:0048729	tissue morphogenesis	485	141	1.0e-05	15	48/+
GO:0031175	neuron projection development	697	251	1.1e-05	32	55/+
GO:0001959	regulation of cytokine-mediated signaling pathway	85	17	1.1e-05	8	8/+
GO:0060271	cilium morphogenesis	107	21	1.1e-05	13	1
GO:0000086	G2/M transition of mitotic cell cycle	147	29	1.3e-05	14	12/+
GO:0006873	cellular ion homeostasis	609	201	1.4e-05	40	24/+
GO:0009410	response to xenobiotic stimulus	152	30	1.5e-05	6	1
GO:0034622	cellular macromolecular complex assembly	553	172	1.7e-05	14	24/+
GO:0030036	actin cytoskeleton organization	421	113	1.7e-05	17	26/+
GO:0031401	positive regulation of protein modification process	741	276	2.0e-05	42	56/+
GO:0006403	RNA localization	139	27	2.0e-05	13	7
GO:0030258	lipid modification	144	28	2.2e-05	13	7
GO:0048878	chemical homeostasis	877	366	2.4e-05	91	37/+
GO:0031497	chromatin assembly	120	23	2.6e-05	8	9/+
GO:0048812	neuron projection morphogenesis	571	180	2.6e-05	18	48/+
GO:0055082	cellular chemical homeostasis	670	233	2.8e-05	52	25/+
GO:0030031	cell projection assembly	214	44	3.5e-05	12	6
GO:0009201	ribonucleoside triphosphate biosynthetic process	71	14	3.7e-05	10	1

Continued on next page

Table 1 – *Continued from previous page*

GOID	GO term	#Genes	#Inter.	p-value	GCC	CG
GO:0009790	embryo development	927	401	3.7e-05	90	96/+
GO:0000723	telomere maintenance	65	13	3.9e-05	7	7/+
GO:0051270	regulation of cellular component movement	470	132	4.0e-05	22	32/+
GO:0060429	epithelium development	606	197	4.2e-05	34	55/+
GO:0048666	neuron development	802	313	4.2e-05	39	60/+
GO:0006486	protein glycosylation	259	56	4.4e-05	7	6
GO:0043413	macromolecule glycosylation	259	56	4.4e-05	7	6
GO:0044724	single-organism carbohydrate catabolic process	132	25	4.6e-05	11	1
GO:0006820	anion transport	321	75	4.8e-05	11	8
GO:0032200	telomere organization	66	13	5.5e-05	7	7/+
GO:0040012	regulation of locomotion	455	125	5.6e-05	17	31/+
GO:0071276	cellular response to cadmium ion	14	5	5.7e-05	5	0
GO:0002449	lymphocyte mediated immunity	179	35	5.8e-05	9	12/+
GO:0048858	cell projection morphogenesis	677	235	6.3e-05	19	49/+
GO:0034329	cell junction assembly	175	34	6.3e-05	5	10/+
GO:1901137	carbohydrate derivative biosynthetic process	665	228	6.8e-05	26	21
GO:0030217	T cell differentiation	162	31	7.0e-05	18	31/+
GO:0006200	ATP catabolic process	73	14	7.4e-05	7	8/+
GO:0019318	hexose metabolic process	247	52	7.8e-05	14	6
GO:0002443	leukocyte mediated immunity	221	45	8.0e-05	10	15/+
GO:0032270	positive regulation of cellular protein metabolic process	823	325	8.3e-05	82	59/+
GO:0006520	cellular amino acid metabolic process	464	128	8.8e-05	20	8
GO:0032990	cell part morphogenesis	689	241	9.0e-05	21	49/+
GO:0016339	calcium-dependent cell-cell adhesion	28	7	1.0e-04	5	0
GO:0018279	protein N-linked glycosylation via asparagine	92	17	1.1e-04	5	1
GO:0006487	protein N-linked glycosylation	98	18	1.2e-04	5	1
GO:0042451	purine nucleoside biosynthetic process	98	18	1.2e-04	12	1
GO:0046129	purine ribonucleoside biosynthetic process	98	18	1.2e-04	12	1
GO:0030163	protein catabolic process	547	165	1.2e-04	17	22/+
GO:0002250	adaptive immune response	211	42	1.3e-04	7	20/+
GO:0009142	nucleoside triphosphate biosynthetic process	75	14	1.4e-04	10	1
GO:0018196	peptidyl-asparagine modification	93	17	1.5e-04	5	1
GO:0006354	DNA-dependent transcription, elongation	110	20	1.5e-04	4	6
GO:0015701	bicarbonate transport	29	7	1.7e-04	5	0
GO:0033344	cholesterol efflux	36	8	1.7e-04	4	1
GO:0007409	axonogenesis	517	150	1.9e-04	11	46/+
GO:0016052	carbohydrate catabolic process	137	25	1.9e-04	11	1

Continued on next page

Table 1 – *Continued from previous page*

GOID	GO term	#Genes	#Inter.	p-value	GCC	CG
GO:0050657	nucleic acid transport	132	24	1.9e-04	12	7
GO:0050658	RNA transport	132	24	1.9e-04	12	7
GO:0051236	establishment of RNA localization	132	24	1.9e-04	12	7
GO:0007005	mitochondrion organization	240	49	2.1e-04	18	10
GO:0071824	protein-DNA complex subunit organization	157	29	2.2e-04	8	10/+
GO:1901701	cellular response to oxygen-containing compound	393	98	2.3e-04	18	24/+
GO:0009100	glycoprotein metabolic process	369	89	2.7e-04	7	12
GO:0019725	cellular homeostasis	754	277	2.7e-04	58	26
GO:0030595	leukocyte chemotaxis	123	22	2.9e-04	8	4
GO:0031349	positive regulation of defense response	230	46	3.0e-04	7	14/+
GO:0002697	regulation of immune effector process	219	43	3.5e-04	9	16/+
GO:0044723	single-organism carbohydrate metabolic process	573	175	4.2e-04	23	13
GO:0034377	plasma lipoprotein particle assembly	17	5	4.3e-04	4	1
GO:0065005	protein-lipid complex assembly	17	5	4.3e-04	4	1
GO:0060326	cell chemotaxis	150	27	4.4e-04	8	6
GO:0042113	B cell activation	169	31	4.5e-04	11	31/+
GO:0030098	lymphocyte differentiation	228	45	4.7e-04	22	42/+
GO:0048167	regulation of synaptic plasticity	91	16	4.7e-04	9	5
GO:0046034	ATP metabolic process	125	22	5.0e-04	12	9/+
GO:0050808	synapse organization	141	25	5.7e-04	5	8
GO:0007091	mitotic metaphase/anaphase transition	46	9	5.7e-04	8	6/+
GO:0042773	ATP synthesis coupled electron transport	60	11	6.1e-04	4	2
GO:0042775	mitochondrial ATP synthesis coupled electron transport	60	11	6.1e-04	4	2
GO:0014070	response to organic cyclic compound	518	148	6.3e-04	20	31/+
GO:0015672	monovalent inorganic cation transport	287	61	6.4e-04	11	2
GO:0008299	isoprenoid biosynthetic process	25	6	7.2e-04	4	0
GO:0035295	tube development	437	113	7.5e-04	18	46/+
GO:0018212	peptidyl-tyrosine modification	203	38	1.0e-03	6	30/+
GO:0001817	regulation of cytokine production	386	93	1.0e-03	21	25/+
